# A first case report of pulmonary hyalinizing granuloma associated with immunoglobulin A nephropathy

**DOI:** 10.1097/MD.0000000000009088

**Published:** 2017-12-08

**Authors:** June Hong Ahn, Jee Seon Kim, Joon Hyuk Choi, Jin Hong Chung

**Affiliations:** a Department of Internal Medicine, Regional Center for Respiratory Disease, Yeungnam University Medical Center; b Department of Pathology, College of Medicine, Yeungnam University, Daegu, Republic of Korea.

**Keywords:** glomerulonephritis, granuloma, immunoglobulin A, multiple pulmonary nodules, respiratory tract

## Abstract

**Rationale:**

Pulmonary hyalinizing granuloma (PHG) is a rare benign disease that has been shown to be associated with the deposition of immune complexes in the lung parenchyma caused by infection or autoimmune diseases. There have been no reports of PHG in association with immunoglobulin A nephropathy (IgAN).

**Patient concerns:**

A 30-year-old woman visited with a 12-month history of dyspnea on exertion and cough that had worsened 1 month before her visit.

**Diagnosis:**

PHG associated with IgAN.

**Interventions:**

Steroid pulse therapy was performed.

**Outcomes:**

The patient was discharged uneventfully.

**Lessons:**

We present a case of PHG presenting as multiple pulmonary nodules mimicking metastatic lung cancer, which was diagnosed using wedge resection of the right middle lobe through video-assisted thoracoscopic surgery.

## 1 Introduction

Pulmonary hyalinizing granuloma (PHG) is characterized by single or multiple pulmonary nodules mimicking lung neoplasms and is diagnosed based on histological findings. To date, PHG has been thought to be associated with deposition of immune complexes in the lung parenchyma caused by infection or autoimmune diseases. Since the first case report by Engleman et al^[1]^ in 1977, few cases have been reported, and most had benign courses with surgery or steroid therapy.

Immunoglobulin A nephropathy (IgAN) is the most common glomerulonephritis in the Western world. IgAN is defined as the predominant deposition of IgA in the glomerular mesangium. Most cases of IgAN present as a primary disease. However, secondary forms of IgAN have been described, including cases associated with liver disease or chronic mucosal inflammation, and those affecting the respiratory tract, gastrointestinal tract, or genitourinary tract. It has been suggested that interaction between mucosal pathogens, environmental antigens, and the mucosal IgA immune system is important in driving pathogenic processes in IgAN.^[2]^

Although PHG is associated with various pathological conditions, we have not found an association of PHG with IgAN. Herein, we describe an interesting case of PHG complicated by IgAN.

## 2 Case report

A 30-year-old housewife who was a nonsmoker visited the outpatient clinic with a 12-month history of dyspnea on exertion and cough that had worsened 1 month before the visit. The patient had allergic rhinitis, but no specific family history, such as cancer or pulmonary disease. Initial examination revealed rhonchi at both lung fields. The patient was alert and vital signs were stable.

**Figure 1:**
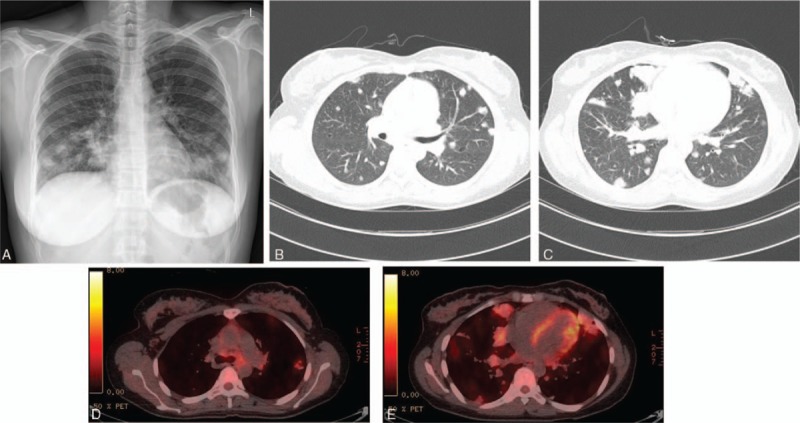
(A) Chest x-ray revealed multiple ill-defined nodules in both lung fields. (B, C) Chest CT revealed multiple randomly distributed lung nodules in both lung fields. (D, E) PET-CT revealed increased FDG uptake in multiple both lung nodules. CT = computed tomography, FDG = fluorodeoxyglucose, PET = positron emission tomography.

Chest x-ray revealed multiple ill-defined nodules in both lung fields (Fig. 1A). Laboratory examinations revealed anemia (11.5 g/dL) and a 5-fold increase in C-reactive protein (2.55 mg/dL; reference, 0–0.5 mg/dL). Blood urea nitrogen (BUN, 9.45 mg/dL) and serum creatinine (0.69 mg/dL) were normal. Serum albumin was normal (3.94 g/dL). Liver function and electrolytes were within normal ranges. Serum measurement of Igs showed elevated total IgG of 2229 mg/dL (normal range, 700–1600), IgA of 546 mg/dL (normal range, 70–400), and IgM of 256 mg/dL (normal range, 40–230). Serum IgG4 was normal (28.4 mg/dL; normal range, 6.1–121.4). Urinalysis showed no proteinuria or hematuria (Table 1). Pulmonary function tests revealed forced vital capacity (FVC) of 2.71 L (75% predicted), forced expiratory volume in the first second (FEV_1_) of 2.36 L (82% predicted), FEV_1_/FVC ratio of 87% (79% predicted), diffusing capacity for carbon monoxide of 17.0 mL/mm Hg/min (76% of predicted), and total lung capacity of 3.61 L (74% predicted).

**Table 1:**
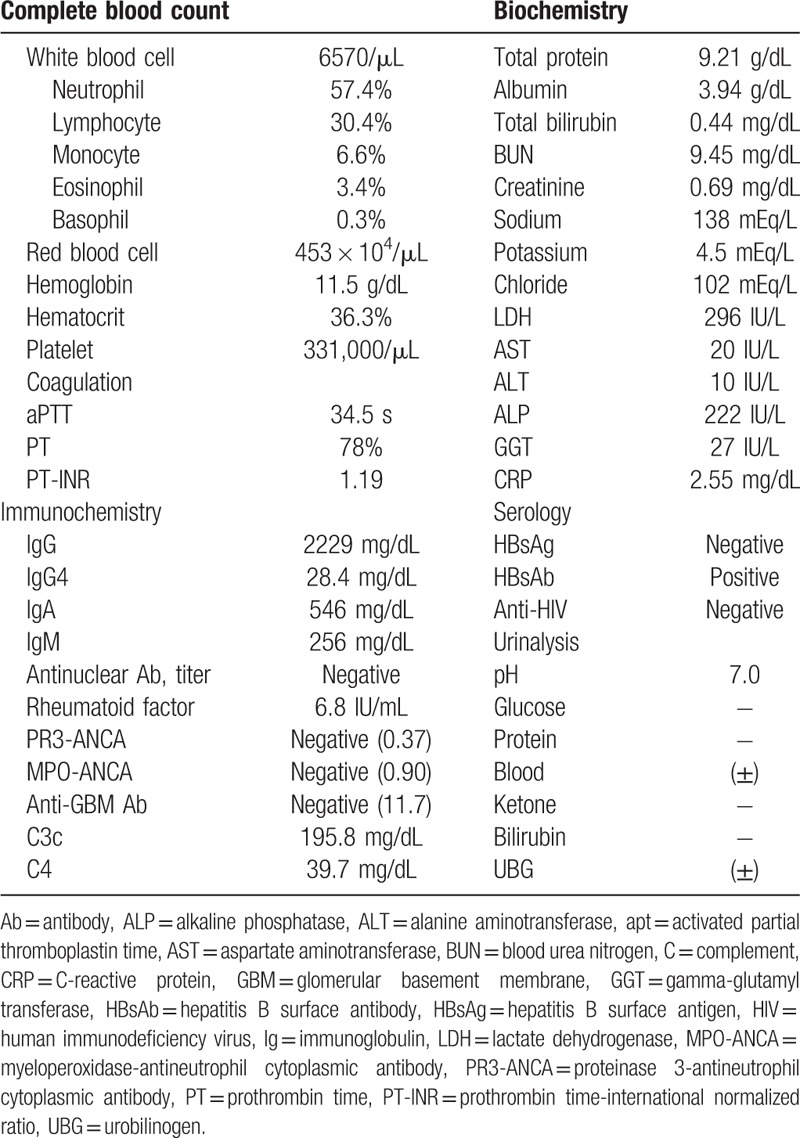
Laboratory data on first outpatient clinic visit.

Contrast-enhanced chest computed tomography (CT) revealed randomly distributed multiple lung nodules in both lung fields with subcarinal lymphadenopathy, suggesting metastatic lung disease (Fig. 1B and C). Fluorodeoxyglucose (FDG) uptake was increased over normal levels at multiple nodules in both lungs, and in the left supraclavicular lymph node upon positron emission tomography CT (PET-CT) (Fig. 1D and E).

Fiberoptic bronchoscopy showed no endobronchial pathological lesions, and a culture of bronchial washing fluids revealed no evidence of tuberculosis or fungal organisms. A fluoroscopy-guided percutaneous needle biopsy was performed, and chronic inflammation with hyalinized material was seen. For definite diagnosis, wedge resection of the right middle lobe was performed through video-assisted thoracoscopic surgery.

**Figure 2:**
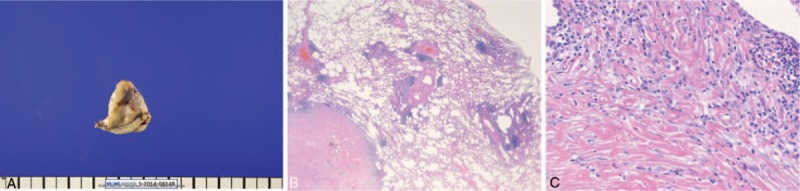
(A) Multiple lung nodules with whitish color were seen. (B) Microscopically, lamellar hyalinized collagen fibers surrounded by lymphocytes are noted (hematoxylin and eosin staining, 10× magnification). (C) Microscopically, lamellar hyalinized collagen fibers surrounded by lymphocytes are noted (hematoxylin and eosin staining, 200× magnification).

Multiple lung nodules with whitish color were seen at the lung surface. The nodules were well demarcated, oval-shaped, and sized 1 to 4 cm (Fig. 2A). Microscopically, they were of lung parenchymal origin and were located in the subpleural area. Histopathological examinations revealed homogenous hyaline lamellae, surrounded by a collection of plasma cells, lymphocytes, and histiocytes, compatible with PHG (Fig. 2B and C). There were no granulomatous lesions. Stains for fungi and acid-fast bacilli were negative. Congo red stain for amyloid was negative. Immunohistochemistry for IgG and IgG4 was performed, and staining for IgG was positive in some plasma cells, although staining for IgG4-positive plasma cells was <1 cell/high-power field.

The patient received prednisone (30 mg/day) for 1 month and a reduced dose of prednisone (15 mg/day) for 1 consecutive month. Follow-up chest CT after 2 months of treatment revealed no interval change compared with the previous chest CT. At that time, the patient complained of peripheral edema caused by the prednisone and was administered low-dose prednisone (10 mg/day) for 2 years, during which time the symptoms and radiological findings were stable.

Recently, the patient visited the clinic with reaggravation of the cough. Chest CT revealed a slight increase in the sizes of multiple lung nodules in both lung fields when compared with the chest CT taken 2 years previously. Proteinuria (4+ according to a dipstick test) and hematuria (1+ according to a dipstick test) were detected through urinalysis. A 24-hour urine study revealed the presence of proteinuria (2586 mg/day). Dysmorphic red blood cells were detected. There was no deterioration of renal function as determined using BUN (10 mg/dL) and serum creatinine (0.93 mg/dL). Serum albumin had decreased (3.19 g/dL). A kidney biopsy was performed.

**Figure 3:**
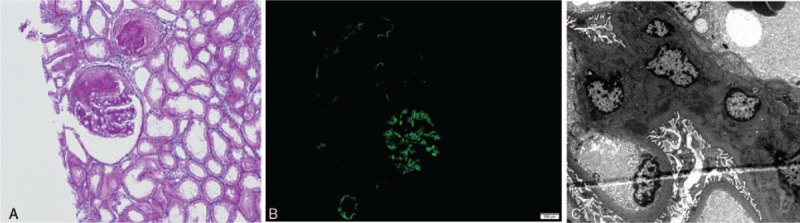
Histological findings of the kidney. (A) PAS staining in light microscopy (100× magnification). (B) Immunofluorescence staining was positive for IgA in the mesangial regions. (C) Electron microscopy revealed dense deposits in the mesangial area. Ig = immunoglobulin, PAS = periodic acid-Schiff.

On light microscopy, there were ∼11 glomeruli, of which 4 glomeruli were globally sclerosed. An additional 3 glomeruli showed segmental sclerosis. Focal tubulointerstitial atrophy with fibrosis (10%) and mesangial hypercellularity were observed upon light microscopy. The hyaline deposits were absent. Vessels were without significant histological abnormalities (Fig. 3A). In the mesangium, immunofluorescence assays revealed 2+ staining for IgA (Fig. 3B). Electron microscopy revealed dense deposits in the mesangial area (Fig. 3C). All these findings were compatible with IgAN.

Steroid pulse therapy (methylprednisolone 1 g/day for 3 days) was initiated, followed by a prednisone supplement (50 mg/day). The patient was also treated with angiotensin II receptor blocker. The patient was discharged, and the dose of prednisone will be gradually tapered. After steroid treatment, urinalysis showed an improvement. Proteinuria (1+ according to a dipstick test) and hematuria (± according to a dipstick test) were detected. There was no change in renal function (BUN, 14 mg/dL; creatinine 1.01 mg/dL). Serum albumin had increased (3.55 g/dL). Neither the size nor number of lung nodules decreased significantly on chest x-ray.

As this study is a clinical case report, no ethical committee approval was required for its conduction, which is in compliance with the institutional and national policies concerning research approvals.

## 3 Discussion

First reported in 1977, PHG is a rare benign disease that is characterized by single or multiple lung nodules mimicking metastatic lung disease.^[1]^ The case described here is the first report of PHG in association with IgAN.

The mean age of PHG presentation is 44.6 years (15–83 years), and male predominance has been reported.^[3]^ Patients usually present with symptoms of cough, dyspnea, and chest pain.^[4]^ About 25% of patients are asymptomatic.^[5,6]^ If the esophagus is compressed by a tumor, dysphagia can develop.^[7]^ Sclerosing mediastinitis can develop if the tumor is located adjacent to the hilum or mediastinum.^[6]^

The etiology of PHG is unknown, but it has been shown to be associated with mediastinal and retroperitoneal fibrosis; autoimmune, infectious, and neoplastic diseases; and thromboembolism.^[3,8,9]^ It has been hypothesized that all these conditions may present essentially the same reactive response to an immunological mechanism.

An association of PHG with IgAN has not been reported to date. The association of PHG with IgAN could be coincidental, but as there is a possibility of immune reaction in the pathogenesis of both diseases, the association is of particular significance.

IgAN has been described in association with liver disease, inflammatory bowel disease, connective tissue diseases, neoplastic diseases, and chronic infections. Such conditions are associated with increased serum IgA levels and IgA-containing immune complexes, and some patients go on to develop IgAN. However, the precise relationship between autoimmune conditions, regulation of IgA synthesis, and development of IgAN remain unclear.^[2]^

PHG usually presents as multiple lung nodules on chest radiography. PET-CT may reveal hypermetabolic activity of the nodules in 60% of PHG patients.^[3]^ Radiologically, few studies have presented multiple pulmonary nodules with high FDG uptake on PET-CT mimicking unique features of metastatic lung cancer, as in this case.^[10]^

Accurate diagnosis of PHG may require an excisional biopsy with appropriate specimens. Pathologically, PHG is characterized by homogenous hyaline lamellae surrounded by a collection of plasma cells, lymphocytes, and histiocytes in a perivascular distribution.^[3]^ The differential diagnosis of PHG includes fungal infections, mycobacterial lung diseases, rheumatoid arthritis, Wegener granulomatosis, sarcoidosis, uveitis, antiphospholipid syndrome, amyloidosis, sclerosing mediastinitis, idiopathic systemic fibrosis, and IgG4-related sclerosing disease.^[11]^ In our case, there was no evidence of bacterial or fungal organisms. We could exclude rheumatoid arthritis, sarcoidosis, and Wegener granulomatosis based on the absence of granulomatous lesions and negative serologic markers. There was no evidence of amyloidosis or sclerosing mediastinitis on histopathological examinations. In our case, serum complement titer, IgG, IgA, and IgM were slightly elevated. However, serum IgG4 was normal, and staining for IgG4-positive plasma cells was negative. Fundoscopic examinations and antiphospholipid antibody testing were not performed because there were no clinical symptoms to suggest uveitis or antiphospholipid syndrome.

The prognosis for PHG is often good.^[3]^ Generally, single lesions are stable for a long time and can be cured via surgical resection,^[6]^ whereas multiple lesions have a worse prognosis. Progressive enlargement of multiple lesions may result in disabling and impaired pulmonary function. Corticosteroids have been reported to provide radiological and symptomatic improvement in most cases.^[3]^ Shinohara et al^[12]^ reported PHG accompanied by laryngeal and subcutaneous involvement that responded well to steroid pulse therapy. In our case, the patient's respiratory symptoms and radiological findings deteriorated despite continuous corticosteroid treatment. As there are no established clinical studies treating progressive and symptomatic PHG despite using corticosteroid therapy, we used steroid pulse therapy after considering previous clinical case reports.

In conclusion, PHG should be considered in patients who present with multiple pulmonary nodules. Radiological studies and optimal histopathological evaluations should be made for definite diagnosis. This case shows an association between PHG and IgAN. Although corticosteroids may improve the natural evolution of PHG in most cases, alternative therapies effective for steroid-refractory PHG should be investigated.
